# Identifying t-cell epitopes of the VP1 capsid protein of human rhinovirus

**DOI:** 10.1186/1939-4551-8-S1-A64

**Published:** 2015-04-08

**Authors:** Cibele Marina Gaido, Belinda J Hales, Peter Le Souëf, Peter Le Souëf

**Affiliations:** 1 The University of Western Australia, Australia; 2 Princess Margaret Hospital for Children, Australia

## Background

The newly identified HRV-C has been linked to more severe symptoms of asthma and an increased risk of prior and subsequent hospital admission. Our research team has recently reported that despite their sequence dissimilarity, the HRV-A and HRV-C species have a high degree of antibody cross-reactivity and the HRV-C specific antibody response is low (Iwasaki et al., 2013). This study aims to identify species-specific and cross-reactive T-cell epitopes for the HRV-A and HRV-C species.

## Methods

In total, 110 overlapping synthetic peptides of HRV-A (genotype HRV-34) and HRV-C (genotype QPM) were purchased from Mimotopes (Vic, Australia) covering the entire VP1 protein of both genotypes. T-cell proliferation was measured by ^3^H-thymidine incorporation. Peripheral blood mononuclear cells (PBMCs) were isolated from the blood of 20 adults from the general population of Perth (WA), with no evidence of current respiratory illness. The PBMCs were cultured in AIM-V media with the individual peptides of HRV-A and HRV-C in triplicate. PHA and PPD were included as positive controls and negative controls contained only media. ^3^H-thymidine incorporation was measured after a 6-day culture and a stimulation index of greater than 2 (calculated by the mean of wells containing antigen divided by the mean of the negative control wells) was considered a positive lymphoproliferative response. Variation on replicate cultures was lower than 20%.

## Results

We have identified four species-specific peptides for HRV-QPM and three for HRV-34 that triggered T-cell proliferation in more than 50% of the tested subjects. Four pairs of cross-reactive peptides were identified for HRV-A and HRV-C that stimulated the cells of more than 40% of the subjects tested.

## Conclusions

To our knowledge, this is the first study to map T-cell epitopes in a genotype of HRV-C and also the first to map the entire HRV VP1 protein. Future HLA typing and in silico studies for other HRV-A and HRV-C genotypes based on the reactive peptides will be conducted. These findings will help to identify differences in the immune recognition of the two most prevalent HRV species and are valuable tools for future vaccine development.

